# Making Headway for Discussions About Concussions: Experiences of Former High School and Collegiate Student-Athletes

**DOI:** 10.3389/fneur.2019.00698

**Published:** 2019-07-05

**Authors:** Anthony Oddo, Ellen O'Conor, Sarah Shore, Mary Piraino, Kyla Gibney, Jack Tsao, Ansley Grimes Stanfill

**Affiliations:** ^1^Department of Pediatrics, University of Tennessee Health Science Center, Memphis, TN, United States; ^2^Department of Neurology, College of Medicine, University of Tennessee Health Science Center, Memphis, TN, United States; ^3^Department of Acute and Tertiary Care, College of Nursing, University of Tennessee Health Science Center, Memphis, TN, United States

**Keywords:** concussion, traumatic brain injury, athlete, sports, adolescent

## Abstract

In order to better understand how to identify and treat student-athletes who experience concussions, better insight into reporting behavior of athletes is needed. This study aims to identify information influencing athletes' attitudes toward reporting their concussions and the perceived trajectory of their recovery both athletically and academically. Twenty-five former high school and collegiate athletes who experienced concussions in a wide variety of sports, organizational levels, and time periods gave insight through structured, qualitative interviews. A number of factors regarding education about concussions, proper diagnosis of concussions, and motivational pressures within high school and collegiate athletics were assessed. Eight major themes were identified regarding the participants' experiences with sport-related concussion: optimism bias, invisibility of the injury, diagnostic barriers, desire to play, external support and pressures, uncertainty of long-term prognosis, generational factors, and protection of future athletes. The findings support that underreporting of concussions among those players interviewed is related to misperceived risk, lack of education, and a struggle between internal and external pressures to play through injury. However, those who did seek medical and academic support, often did receive the necessary aid.

## Introduction

Despite mandated concussion education programs and increasing media attention to the risks of playing while concussed, many high school and collegiate athletes continue to misrepresent and underreport concussion symptoms (1). Several factors lead to this underreporting behavior. Clinical diagnosis of concussion is highly dependent on self-reported symptoms, including subjective neurological signs such as headaches, confusion, and amnesia. In addition, athletes' current understanding of concussion often does not coincide with the accepted medical definition (2, 3). Motivational issues also contribute to the underreporting of symptoms and continuing to play through injuries (4).

Some have suggested that targeted concussion education may increase reporting, but there is also evidence to suggest that such efforts may not equate to increased reporting; possibly due to some of the reporting motivation issues as previously mentioned (4–8). High school and collegiate athletes continue to have some of the highest rates of unreported concussion (9–11).

But concussive injuries during adolescence and early adulthood can have serious medical repercussions for the developing brain, though most notably in the subacute phase of the injury (12, 13). Although removal from play decisions are of immediate importance post-injury, in the absence of clear guidelines for return to classes, some student athletes may suffer academically as well. Few studies have been done to establish more effective removal/return to play or return to school guidelines (14, 15).

The purpose of this work was to gain information about reasons that they did or did not report their injury and the perceived trajectory of recovery athletically and academically. The sample was not limited to those that were more recent athletes, allowing analysis of the effect of increasing media attention and concussion education efforts on recent experiences and comparison to older individuals. The goal of this work is to enhance the knowledge of these factors in order to design effective educational interventions that will increase reporting behavior for these athletes and to establish more effective guidelines for supporting a concussed student athlete during their recovery process.

## Methods

### Design

A retrospective, survey study was performed and individual, face-to-face, structured interviews were conducted with former high school and college student athletes that sustained a sports-related concussion while playing organized sports. The interview responses were reviewed to identify key themes. These themes were further analyzed as they may have or have not related to the demographic background of the participants in order to reveal any trends, consistencies, or differences in the athletes' concussion experiences and reporting behaviors.

### Participants

Participants were recruited by university email announcements to faculty, staff, and students, and by fliers placed on campus and in local coffee shops and gyms. Inclusion criteria consisted of English-speaking, over the age of 18, having participated in organized athletics during their high school and collegiate career and having sustained one or more sport-related concussions through this participation. Additionally, the former athletes must have access to an email account for initial contact and scheduling purposes. Exclusion criteria was a history of severe head trauma or neurologic disease (e.g., headache disorder/migraines, mood disorder, epilepsy/seizure disorder, etc.) prior to the experience of sports related concussion. The study staff explained the study procedures, risks, benefits, and alternatives to participation, and the participants signed an informed consent document. This protocol was approved by the Institutional Review Board (IRB#16-04937-XP).

### Procedures

All participants were interviewed face to face with a single examiner in an office suite. Interview questions were read verbatim by the interviewer and are provided in Table 1. Individual interviews' content validity was established at the end of each session. Then, the interviewer verbally summarized the subjects' comments. Subjects were allowed to clarify any comments and to add any further thoughts on their concussion experience. Each interview was recorded by an audiotape recorder by the interviewer. All audiotapes were transcribed and verified by a second member of the study staff.

**Table 1:** Structured interview script.

*The first set of questions ask you to describe the circumstances of your concussion. If you have had multiple concussions, please answer the questions for each instance of injury*.	
1.	Please tell me about the type of athletics you participated in, how it was organized by your school (such as club or varsity athletics) and the sport or sports you were playing when you experienced a concussion.
2.	Please tell me general details about the size, location, and athletic division ranking of the school that sponsored these athletic activities, but do NOT tell me the name of the specific school.
3.	About how old were you when you experienced your concussion or concussions? What year or years were you in school?
4.	How long has it been since then?
5.	How old were you when you first started playing this sport?
6.	Prior to your concussion in high school or college, were you aware of the risk of head injury in playing your particular sport or sports?
7.	Would you rate your particular sport or sports as being high risk, medium risk, or low risk for head injury?
8.	What education have you received regarding concussions? Would you say that this education has come from your school or schools in general, your coaches, your parents, the media, or some other source?
9.	Do you feel that there is anything that could have prevented your concussion (such as different safety equipment, more or less intense practices, etc.)?
10.	What were the circumstances of your injury?
11.	How was your injury discovered? Did you lose consciousness?
12.	What other kinds of symptoms do you remember having? How long did these symptoms last, and how bothersome were they in your daily life?
13.	How were you evaluated immediately after your injury?
14.	Did you receive any medical care or other special evaluation in the days following the injury?
15.	Did you receive any additional information about concussion in the days following the injury?
16.	If you reported the event, did you feel comfortable going to your coach or trainer? Would you say that your teammates, or others in your sport felt the same way?
*The next set of questions ask you to describe the recovery after your concussion*.	
17.	When were you able to return to play after your injury? Were there any restrictions once you were allowed to return to practice or competition?
18.	Did you feel that there were any repercussions from your coach or trainer because of your injury? If so, please describe.
19.	Were you concerned about repercussions for athletic or academic scholarships because of your injury? In what way?
20.	How long do you feel that full recovery took?
21.	What changes did you, your professors or teachers, or school administrators have to make to ensure your academic success during the recovery period?
22.	Were there any other changes you had to make in your daily life in the time right after your concussion?
23.	What kind of social support network did you have during your recovery? Who were the members of that network? What did this network specifically help you with during the recovery period?
24.	Did you have to withdraw or drop any courses related to your injury?
25.	Did you feel that your academics were affected by your concussion? If so, how were they affected?
26.	Do you have any lasting effects now that you feel are due to your concussion or concussions? Would you say that these are problems that you have daily, occasionally, or rarely?
27.	Have you ever been diagnosed with post-concussion syndrome or any other health issues related to your concussion?
28.	Do you feel that the current media attention surrounding concussion (such as the Concussion movie featuring Will Smith, and litigation in NCAA and NFL athletes) has changed any of your perceptions or feelings about your past injury? In what way?
29.	Do you have any other thoughts about sports-related concussion that you would like to share with us?

*Before we leave today, I am going to briefly summarize what I feel are the most important themes of your comments. Please stop me or correct me if I have misunderstood anything that you have said. [Insert summary of the subject's comments and allow for subject agreement]*.

### Analysis

The data analysis team followed the method that is described in the “Long Table Approach” (15). Briefly, 6 of the authors individually read the transcripts multiple times and summarized the main points and key quotes from each interview. From these items, individual team members identified themes in the data. Then the team met as a group, debated and provided evidence for each of the identified themes until a consensus was reached. From this iterative process, the research team developed major and minor overarching themes and recommendations for factors to be considered when designing future guidelines for student athletes experiencing a concussion.

## Results

### Demographic Characteristics of the Study Participants

The study cohort included 25 former high school and college athletes. Demographics of these participants are described in Table 2.

**Table 2:** Characteristics of the sample.

**Characteristic**	**Category**	***n***
Gender	Male Female	21 4
Time since concussion	< 10 Years > 10 Years	13 12
Sport played	Football Basketball Soccer Softball/Baseball Rugby Cheerleading Polo Running Roller derby Wrestling	8 5 4 4 3 1 1 1 1 1
Type of school	High school College Other	13 14 2
Organizational level	High school	13
	Junior varsity	3
	Varsity	8
	Collegiate	17
	Intramural/Club	6
	Junior college	1
	Division I	1
	Division II	5
	Division III	4

*N.B. Subtotals of individual categories may not add up to 25, as some participants may have reported concussions during involvement in multiple sports, at multiple ages, or in multiple organizational levels*.

Eight major themes were identified regarding the participants' experiences with sport-related concussion: optimism bias, invisibility of the injury, diagnostic barriers, desire to play, external support and pressures, uncertainty of long-term prognosis, generational factors, and protection of future athletes. A list of these themes can be found in Table 3, and each of these is described below.

**Table 3:** Interview response themes and supporting quotations.

**• Optimism bias**
° *“It just happened out of nowhere… I never thought that it would happen to me.”*
° *“My particular accident was a pretty random accident.”*
**• Invisibility of the injury**
° *“You know I didn't have a cast. I didn't have a broken leg.”*
**• Desire to play**
° *“I wanted to be out there every single play, regardless of how I felt.”*
**• Diagnostic barriers**
° *“My coaches knew to ask me repetitively, “Are you okay?” and I just didn't know the correct answer. I just didn't know to say “No.” I just felt like I had to say “Yes.””*
° *“You just got hit in the head, so you are not thinking real clearly. You need other people to watch out for you.”*
**• External support and pressures**
° *“I almost felt like it was held against me-that I had to miss a week because I had a concussion. It wasn't my fault.”*
° *“The teachers and professors were very helpful…they gave me a good program to get me back in.”*
**• Uncertainty of long-term prognosis**
° *“I don't think we thought much about head injuries or being turned into a vegetable or something from it.”*
° *“It would be useful to make it more clear like we do for smoking, like what is the risk…they give you a very vivid picture of the down side, and I don't feel like you get that at all…”*
**• Generational factors**
° *“I'm kind of jealous of the medical care that players get now compared to what my era of players got. So, I am really jealous about…what kind of impact that might have had on my life.”*
° *“It was “Rub some dirt on it, get back out there, let's go!””*
° *“I felt okay going to the trainer because that's what he's there for.”*
**• Protection of future athletes**
° *“Some of the things that happened to me, I don't want them to happen to other athletes.”*
° *“I think the more restrictions put on it, the better.”*

#### Optimism Bias

Participants expressed varying levels of understanding regarding the chances of experiencing a sports-related concussion. However, those who were familiar with the risk of concussion displayed an ignorant awareness, with numerous athletes stating that they did not believe that they themselves would sustain a concussion: “It just happened out of nowhere… I never thought that it would happen to me.”

Eighty-four percent (21/25) of subjects felt that concussions were not preventable, often explaining that their own experiences were out of their control: “My particular accident was a pretty random accident, and there wasn't anything necessarily that could have been done.”

#### Invisibility of the Injury

Student athletes often reported that because of the lack of visible signs or symptoms, it was easy for the concussion to not be recognized not only in diagnosis, but also in the post-injury recovery setting: “It's because it is such an invisible injury. You know I didn't have a cast. I didn't have a broken leg.”

This lack of a visual indicator for injury led many participants to describe circumstances where coaches and peers did not fully comprehend the severity of the concussion. These circumstances led to feelings of isolation. One participant said “My coaches didn't understand, my trainer didn't understand, my teammates certainly didn't understand. They don't know anything about head injuries…I felt like I was on my own.”

#### Desire to Play

Some athletes utilized the invisibility of concussion to allow them to participate in sports while injured, as depicted by this remark: “I didn't really have any limitations because I was silent about [my concussion].”

Participants shared feelings that they were unaware of the seriousness of their injury or simply did not care about the consequences of continuing to play. One commented “I was asked if I was all right, and I just said I was, and did not say anything further.”

Other justifications for not reporting concussion symptoms included wanting to keep their starting role and sensing an obligation as a team player, depicted by this response: “There is this internal pressure…because I didn't want to let my team down… I wanted to be out there every single play, regardless of how I felt.”

#### Diagnostic Barriers

As previously mentioned, concussions pose unique barriers for diagnosis. If a head trauma is not witnessed or if no loss of consciousness takes place, recognition and diagnosis of a concussion is reliant only on an athlete's self-reported symptoms. Many athletes often did not recognize their symptoms and continued to play: “I could tell something was wrong because I couldn't remember the play…I didn't know what to do. I had to ask a teammate what I did on each play.”

Many symptoms that participants reported (such as headaches, fatigue, or light sensitivity) can be difficult to recognize by coaches or medical staff. Several former student athletes stressed the importance of someone else recognizing their concussion, as described by the following response: “You just got hit in the head, so you are not thinking real clearly. You need other people to watch out for you.”

#### External Support and Pressure

Participants gave a variety of responses in terms of describing external support and pressure in sharing their symptoms after experiencing a concussion. Some described a continued lack of understanding from coaches. One participant stated, “I almost felt like it was held against me-that I had to miss a week because I had a concussion. It wasn't my fault.” Even with athletic staff who recognized a concussive injury, one participant shared their internal struggle to balance pressures from coaches: “My coaches knew to ask me repetitively, ‘Are you okay?' and I just didn't know the correct answer. I just didn't know to say ‘No.' I just felt like I had to say ‘Yes'.” Also, a sense of comradery among teammates led to athletes playing through injury both in support of their peers, as well as out of fear of judgment from their peers if they were to sit out.

The former student athletes did consistently report that they received many academic accommodations after reporting their concussion, whether it meant missing class, delaying tests, or even receiving one-on-one tutoring. One participant describes their academic experience after suffering a concussion: “The teachers and professors were very helpful…they gave me a good program to get me back in.”

#### Uncertainty of Long-Term Effects

Former athletes shared their concern about the increased media attention for concussions, and the potential for more lasting effects post-concussion. The recent studies on NFL football players have often been highlighted (16), although there may be dangers in overstating some of these claims (17). One person said, “You know we got injured all the time in football, but I don't think we thought much about head injuries or being turned into a vegetable or something from it.” Another participant alluded to the greater certainty of risk with other disease processes and behaviors “It would be useful to make it more clear like we do for smoking, like what is the risk…they give you a very vivid picture of the down side, and I don't feel like you get that at all, especially if you are doing it at my level.” While many participants were very competitive in high school and collegiate athletics, it is currently unclear what the actual risk of long-term sequelae is for them.

Many interviewees voiced discontent with the knowledge of their coaches and medical staff when they were playing. One participant stated the following: “When they say, ‘You got a concussion,' you just shook it off. You just got up and you got going. The medical care was very poor and the knowledge of the long-term was nonexistent.”

#### Generational Factors

Interviewees displayed a dichotomy in responses between those that experienced their concussion greater than 10 years ago, and those that experienced their sports-related concussion within the past 10 years. Twelve of the 25 interviewees sustained a concussion over 10 years ago, with 5 having sustained their concussions more than 20 years ago. Common trends for these older individuals suggested a general lack of education and awareness regarding concussion risks. One interviewee, who sustained a sports-related concussion over 20 years ago stated, “I'm kind of jealous of the medical care that players get now compared to what my era of players got. So, I am really jealous about…what kind of impact that might have had on my life.” These injured athletes returned to play almost immediately, with very few mentioning any type of restrictions. One older participant explained, “It was ‘Rub some dirt on it, get back out there, let's go'!”

In comparison, players who sustained a concussion within the last decade reported a more positive outlook regarding these same questions. Younger participants mentioned receiving education on concussions prior to injury, as well as feeling more comfortable reporting the injury to athletic staff members. One participant commented, “I felt okay going to the trainer because that's what he's there for.” Athletes also described receiving formal evaluations from athletic trainers and/or physicians following the injury, and underwent a more progressive, delayed return to play.

#### Protection of Future Athletes

The majority of participants across all age groups expressed support for the strategies being implemented to address concussive injuries. Several individuals reflected on their own injuries when discussing the protection of future athletes, such as one interviewee who commented: “Some of the things that happened to me, I don't want them to happen to other athletes, and having the media attention… and the litigations that are happening I think have helped educate many parents, coaches, trainers, everybody that is involved within organized sports, to be more aware and attentive to what needs to be done.”

Various participants shared feelings of concern for their own children playing certain sports that they judged to be at high risk for concussion, such as the parent who said, “I will probably never let my kids play football.” Other former athletes advocated for more stringent concussion-prevention protocols and rules, with one person stating, “I think the more restrictions put on it, the better.”

## Discussion

Prior qualitative studies have explored student-athletes' education regarding concussion, past or present personal experiences of concussion, and support of concussed individuals as they return to school. From this work, it becomes very apparent that healthcare providers, coaches, athletic trainers, teachers, and parents should learn more about concussions, understand that they can occur in all sports, and recognize signs or symptoms of injury (18). The current study is consistent with, but adds to, this prior literature. The themes identified reveal that multiple barriers still exist for athletes to recognize, report, and safely recover from concussions. Overall, participants described several ideas that are amenable to future intervention, leading to better concussion reporting behaviors.

Foremost, educational interventions must be done to help athletes recognize a concussion and its associated symptoms. Even though the participants were responding to a call for individuals that had experienced concussion, there was still confusion on what constituted such an injury. One participant asked “When you say concussion what's the definition? Are you talking about where I black out and had to be woken up? Or are you talking about bell-ringers? What is the definition of a concussion?” This lack of clarity occurs within the context of the diversity of concussion symptoms (including sleepiness, headache, photosensitivity, and short-term memory loss). Often, no two concussions are exactly alike, even when experienced by the same athlete. Until there is a truly objective and accurate assessment method (such as a concrete and measurable biomarker), medical personnel remain dependent on reported symptoms.

Concussion diagnosis remains subjective, and so there must be self-advocacy of the athlete to get the care they require. Beyond being able to recognize appropriate symptoms of concussion, athletic training staff and coaches must remain approachable and supportive for an athlete to report an injury that may have not been witnessed. Ultimately, the burden of reporting currently still falls to the athlete, not only during the acute period, but also for seeking out appropriate medical follow up. Nearly all participants stated that they did not receive additional medical care or education regarding concussion in the days following injury. The participants who did seek medical attention reported receiving a thorough work up, one which included radiological imaging and observational stays in the hospital. Of note, some younger athletes did refer to new protocols in place that included re-evaluation by medical professionals periodically after their initial assessment at the time of injury.

In the area of academics, student-athletes reported concussions to have little impact on their academics once certain accommodations were made. School professionals see other barriers. For instance, school nurses and athletic trainers report that legislative efforts supported their management of return-to-play and return-to-school, but barriers still included lack of educator knowledge and inconsistent care from physicians (19), as well as established social norms for recognition and underreporting of concussions (20). Still, none of the interviewees provided any indication of educational staff opposition to academic workload modifications. Only one athlete reported a significant negative impact on their academic performance even after modifications were made. Currently, there is no legislative enforcement for clearance in returning to classwork.

Overall, there still remains a clear barrier in that many athletes are simply not motivated to report symptoms of concussion. When educational efforts about concussion safety are directed at the athletes themselves, one study reported an increased desire to avoid collisions, but behaviors for reporting concussions that occur may still be unchanged (21). Indeed, many athletes want to play despite any potential cost to their own health, an attitude which creates ethical conflicts between the needs of the player, the needs of the team, and the goals of the medical community (22). Kuhn has shown that participation in some sports, such as football, is a risk factor for not disclosing injury (23). In certain sports, there can be a cultural trend to “tough it out.” Another factor driving this desire to play is the comradery felt among teammates, and continued play may happen to avoid being isolated from this social structure (24). Adolescence is commonly noted for increased peer influence, especially regarding risk-taking behaviors, however, other studies demonstrate peer influence on positive behaviors as well (25, 26). Future interventions would be wise to capitalize on this relationship in order to increase reporting behavior, perhaps by focusing on the safety of the team or peer-group as a whole. Healthcare providers could advocate for this developmental consideration to be made in educational efforts. Perhaps a push for “buddy-system” type efforts could increase reporting compliance. Such a peer-based approach could aid in targeting the motivational struggle in reporting concussions, and over time improve social customs that could lead to safer behaviors.

Regarding limitations of the study, despite efforts to recruit off-campus, many participants had university affiliations and higher educational backgrounds which limited the diversity for these demographics. Furthermore, the respondents were mostly male (21/25), which limits the generalizability of these findings for female athletes that sustain a concussion. Also, all former athletes experienced concussions during team athletics, so these responses may have limited applicability to individual sports. These features of the recruitment method and sample limit generalizability. One advantage of this method was that it allowed both athletes from club and intramural level sports to be included in this study, which often do not have formal coaches and/or training staff present during activities. These subgroups were included in hopes of creating a diverse array of athletic backgrounds, and they emphasize the barriers of recognizing and treating concussion in these various settings.

As the study is within the qualitative field of research, the themes identified are subject to the bias of those analyzing the interview responses. This bias is somewhat unavoidable. Still, the authors minimized potential bias by repeating analyses with each member of the team, each of whom is familiar with Long Table Approach, and then iteratively and collectively agreeing upon themes that were reflective of subject responses.

There are several avenues of future inquiry that are possible based upon these results, and which could influence effective interventions toward increasing reporting behavior. First, continued inquiries into the motivational aspects of reporting behavior remain relevant, and this study should ideally be repeated with a larger study population, as the current group only had 25 individuals. A larger group would be more representative of a collective concussed population, and, therefore, would be helpful in general education and demographic-specific education, alike. More specifically targeted recruitment (i.e., for a particular sport) would likewise enhance educational efforts. Further studies should be conducted involving: evaluations of the risk-taking behavior of those athletes who continue to play while concussed (despite adequate knowledge of the risks); the nature of coach-athlete relationships and their changing influences on reporting behavior; and the influence of team vs. individual sports on reporting behavior. In addition, there is little investigation regarding appropriate timing for a return to academics for student athletes, nor modifications to academic workload. Ultimately, the search for an objective and definitive biomarker of concussion is ongoing and should be continued (27).

Interventions recommended based on these current findings include: (1) continuing student-athlete education on recognizing concussion symptoms in themselves and their peers, (2) encouraging coaching staff and athletic trainers to create a team culture where athletes feel comfortable reporting their injuries and symptoms after injury, (3) stressing to all members of the athletic community, including athletes, family members, coaches, and training staff the value of player safety over player performance, (4) stressing the importance of requesting academic support after a concussion and (5) continuing research both in barriers and facilitators of reporting concussion symptoms as well as objective measures, such as biomarkers.

Many former athletes were aware of the potential for concussions in athletics, yet they felt that such injuries would still not happen to them. Once a concussion did occur, many athletes did not report their injury due to lack of education regarding concussions, an internal drive to play and/or external pressure to play by coaching staff. From the individuals captured, academic needs were met, however, this study included participants from a relatively well-educated background. Participants continued to be plagued by the lack of information regarding long term effects of concussions, and as more information is revealed, many of these former athletes support increased awareness and steps taken to protect future athletes.

## Ethics Statement

This study was carried out in accordance with the recommendations of UTHSC Institutional Review Board with written informed consent from all subjects. The study staff explained the study procedures, risks, benefits, and alternatives to participation, and the participants signed an informed consent document in accordance with the Declaration of Helsinki. This protocol was approved by the Institutional Review Board (IRB#16-04937-XP).

## Author Contributions

JT and AS contributed conception and design of the study. EO performed the structured interviews. AO, EO, SS, MP, KG, and AS performed qualitative analysis. AO wrote the first draft of the manuscript. AO, EO, SS, MP, KG, and AS wrote sections of the manuscript. All authors contributed to manuscript revision, read and approved the submitted version.

### Conflict of Interest Statement

The authors declare that the research was conducted in the absence of any commercial or financial relationships that could be construed as a potential conflict of interest.
